# Supplementary material for: M13 phage grafted with peptide motifs as a tool to detect amyloid-β oligomers in brain tissue
Source: Commun Biol. 2024 Jan 27;7:134. doi: 10.1038/s42003-024-05806-5 (PMC10821927; doi:10.1038/s42003-024-05806-5)
Supplement: Supplementary file 2 — Supplemental information [file 42003_2024_5806_MOESM2_ESM.pdf]

SUPPLEMENTARY INFORMATION

M13 phage grafted with peptide motifs as a tool to detect amyloid-β oligomers in brain tissue

Ivone M. Martins*[,1,2,3,4], Alexandre Lima*[,1,2,4], Wim de Graaff[4], Joana S. Cristóvão[5,6], Niek Brosens[,4], Eleonora Aronica[7], Leon D. Kluskens[1,2,†], Cláudio M. Gomes[5,6], Joana Azeredo[1,2] & Helmut W. Kessels[3,4]

[1] CEB- Centre of Biological Engineering, University of Minho, 4710-057 Braga, Portugal

[2] LABBELS – Associate Laboratory, Braga/Guimarães, Portugal

[3] Netherlands Institute for Neuroscience, Amsterdam, the Netherlands

[4] Swammerdam Institute for Life Sciences, University of Amsterdam, Amsterdam Neuroscience, Amsterdam, the Netherlands

[5] Biosystems & Integrative Sciences Institute, Faculdade de Ciências, Universidade de Lisboa, Lisboa, Portugal

[6] Departamento de Química e Bioquímica, Faculdade de Ciências, Universidade de Lisboa, Lisboa, Portugal

[7] Amsterdam UMC location University of Amsterdam, Department of (Neuro)Pathology, Amsterdam Neuroscience, Amsterdam, the Netherlands

* These authors contributed equally

† Deceased

email: ivone.martins@ceb.uminho.pt; h.w.h.g.kessels@uva.nl

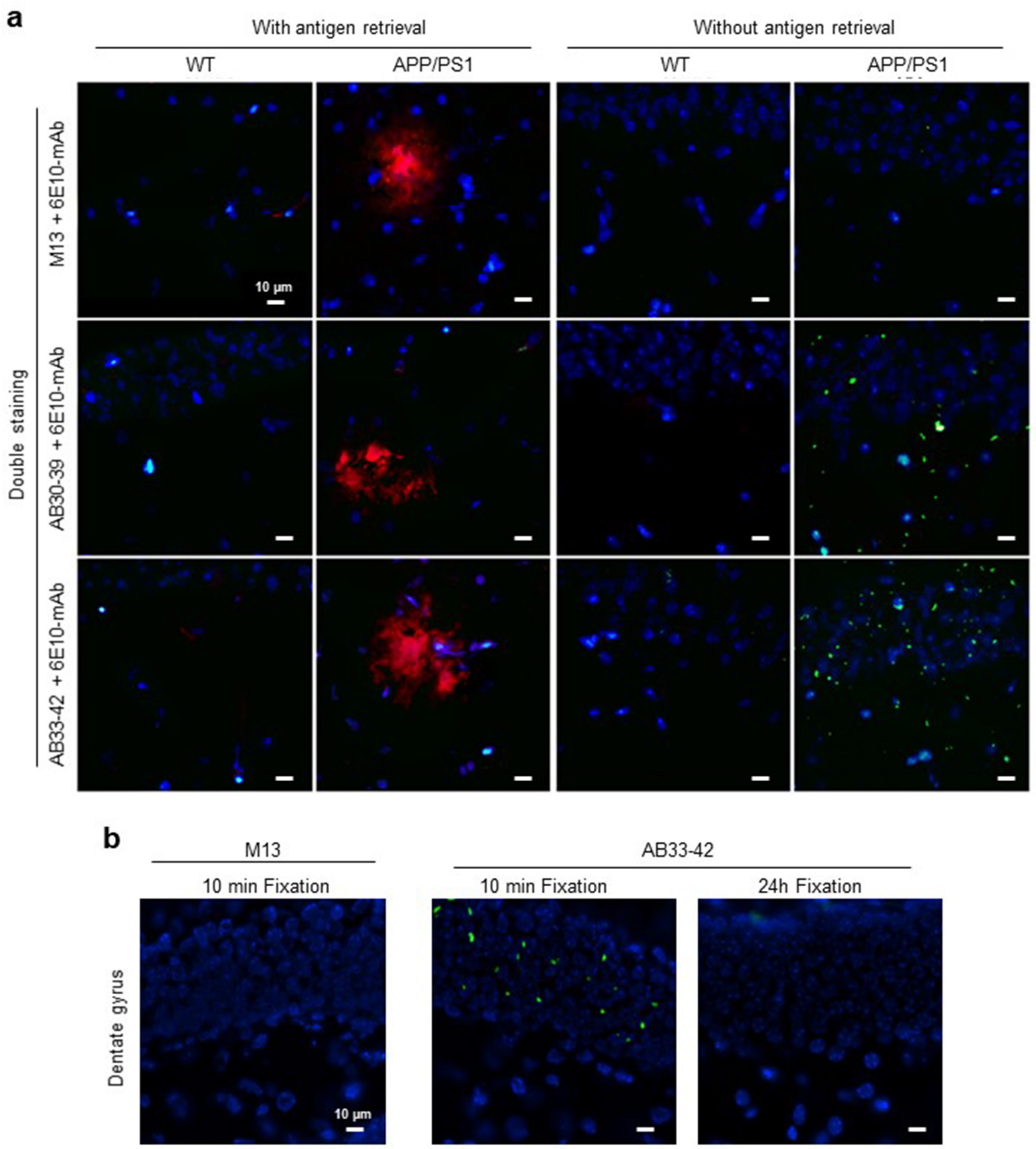

Supplementary Figure 1. Thermal denaturation treatment or extended fixation of brain tissue prevents detection of Aβ-oligomers by phages. a Brain tissue samples from WT and APP/PS1 mice (10-month-old) were incubated with both 6E10 antibodies (red), with $1x10^8$ pfu ml^{-1} M13, AB30-39 and AB33-42 phages (green), and with DAPI (blue). Staining in hippocampal CA1 region is shown. Samples that were treated with thermal denaturation for antigen retrieval (left) were efficiently stained for amyloid-plaques with 6E10, but not for Aβ-oligomers with AB30-39 and AB33-42 phages. Samples without antigen retrieval (right) showed efficient staining for Aβ-oligomers with

AB30-39 and AB33-42 phages, but not for amyloid-plaques with 6E10. **b** Brain tissue samples from APP/PS1 mice (3-month-old) were incubated with 1x10^8 pfu ml^{-1} M13 and AB33-42 phages (green) and with DAPI (blue). Staining in hippocampal dentate gyrus region is shown. Whereas samples were fixated for 10min with paraformaldehyde (left) showed AB33-42 signal, those that were fixated for 24h (right) did not.

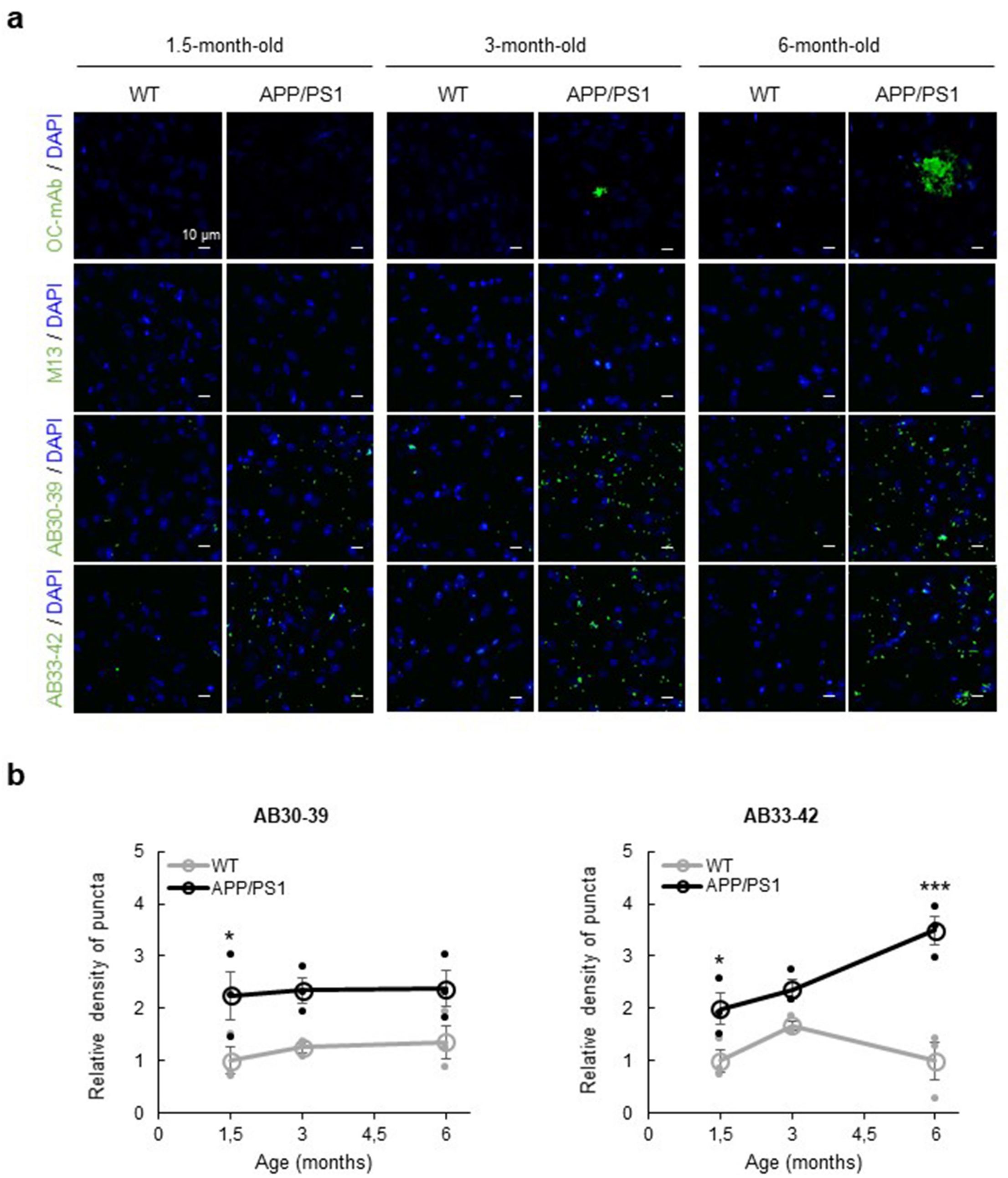

Supplementary Figure 2. Accumulation of Aβ-oligomers in primary somatosensory cortex of APP/PS1-transgenic mice. a Representative immunostaining examples of primary somatosensory cortical tissue from 1.5-month-old, 3-month-old and 6-month-old wild-type (WT) and APP/PS1-mice with OC-mAb or phages (green) and DAPI (blue). **b** Density of ≤1µm puncta density (relative to 1.5-month-old WT) in CA1 from wild-type (grey, n=3) and APP/PS1 mice (black, n=3) after exposure to AB30-39 (left) or AB33-42 (right). Data are mean ±SEM. Statistics: Students *t*-test with Šidák correction for multiple comparisons, *p<0.05, **p<0.01 and ***p<0.001.

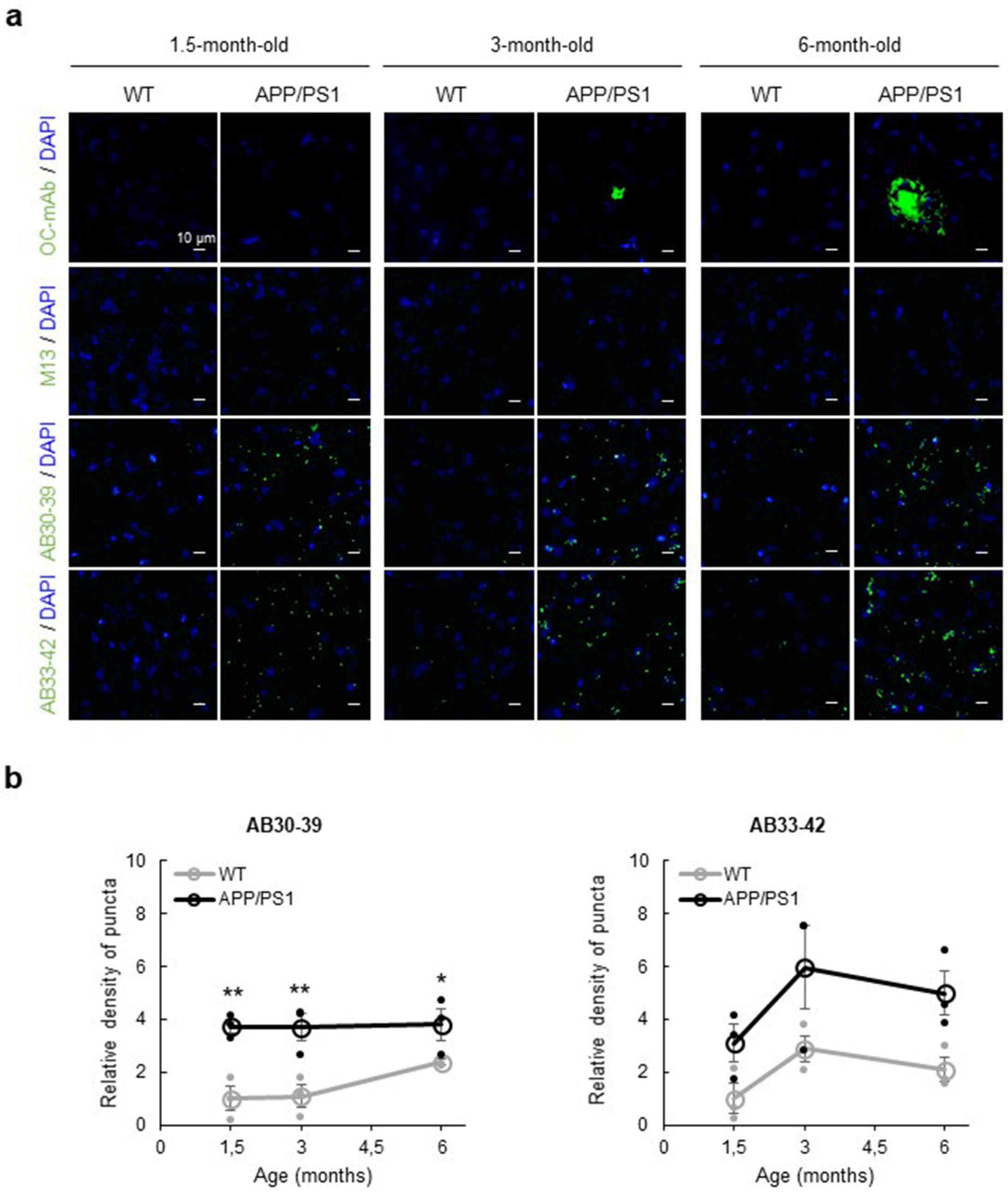

Supplementary Figure 3. Accumulation of Aβ-oligomers in entorhinal cortex of APP/PS1-transgenic mice. a Representative immunostaining examples of entorhinal cortical tissue from 1.5-month-old, 3-month-old and 6-month-old wild-type (WT) and APP/PS1-mice with OC-mAb or phages (green) and DAPI (blue). **b** Density of ≤1μm puncta density (relative to 1.5-month-old WT) in CA1 from wild-type (grey, n=3) and APP/PS1 mice (black, n=3) after exposure to AB30-39 (left) or AB33-42 (right). Data are mean ±SEM. Statistics: Students *t*-test with Šidák correction for multiple comparisons, *p<0.05, **p<0.01 and ***p<0.001.

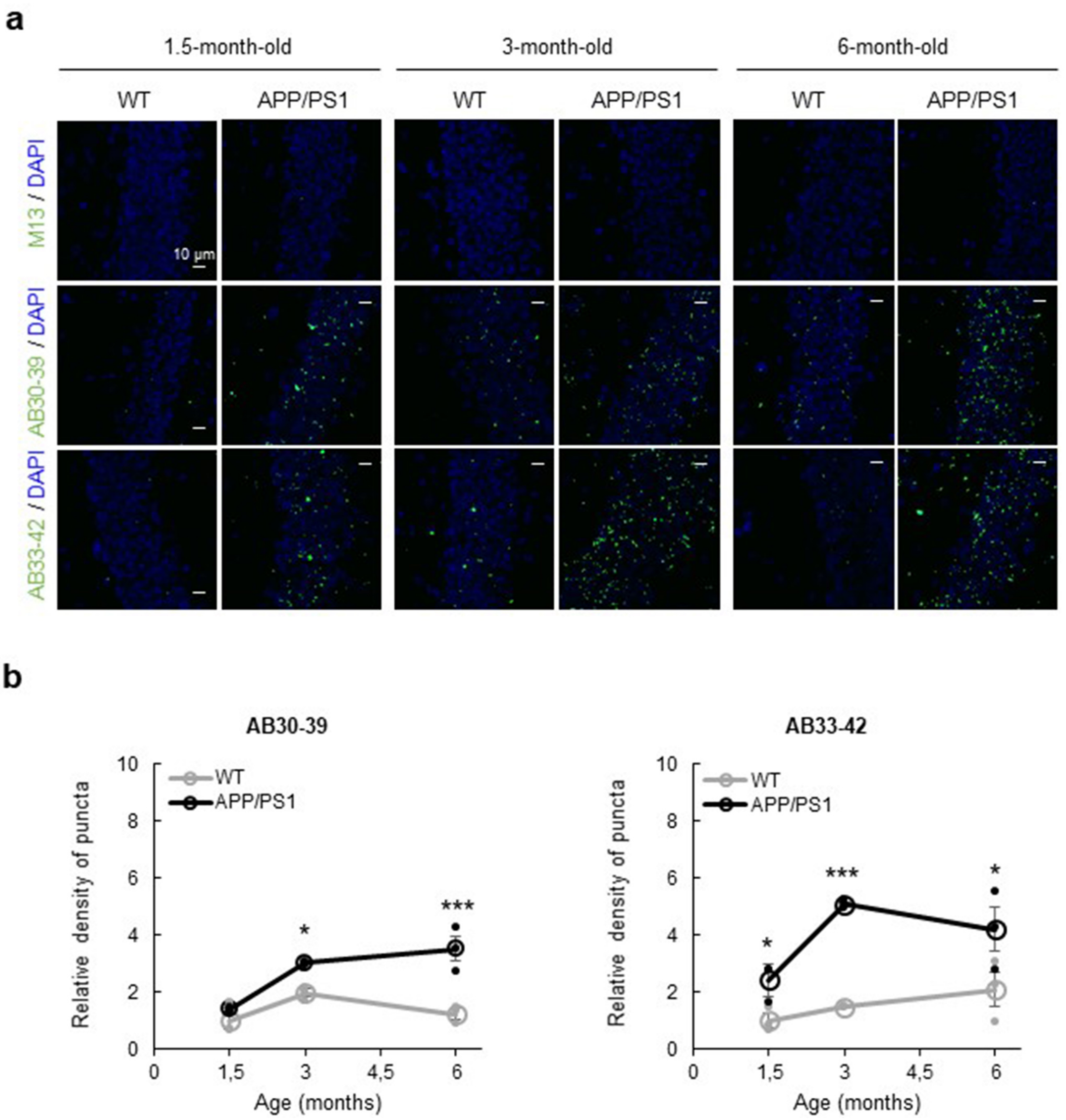

Supplementary Figure 4. Accumulation of Aβ-oligomers in dentate gyrus of APP/PS1-transgenic mice. a Representative immunostaining examples of dentate gyrus hippocapal tissue from 1.5-month-old, 3-month-old and 6-month-old wild-type (WT) and APP/PS1-mice with OC-mAb or phages (green) and DAPI (blue). **b** Density of ≤1µm puncta density (relative to 1.5-month-old WT) in CA1 from wild-type (grey, n=3) and APP/PS1 mice (black, n=3) after exposure to AB30-39 (left) or AB33-42 (right). Data are mean ±SEM. Statistics: Students *t*-test with Šidák correction for multiple comparisons, **p*<0.05, ***p*<0.01 and ****p*<0.001.

Supplementary Table 1. Brain tissue samples from human donors. Thal phase based on the detection of immunopositive amyloid in cortical and subcortical areas: phase (3) - brainstem/midbrain.

Sample	Sex	Age (years)	Thal Phase	AD Change*
Control	Female	76	-	$A_0B_0C_0$
	Male	86	-	$A_1B_1C_0$
	Female	81	-	$A_1B_1C_0$
AD patients	Female	73	3	$A_3B_3C_3$
	Female	89	3	$A_3B_2C_2$
	Female	79	3	$A_3B_3C_2$

*AD neuropathological change (Neuro Change) was evaluated trough ABC score: A - thal Phase for Aβ plaques; B - Braak neurofibrillary tangles score; C - CERAD neuritic plaque score.
