# Supplementary material for: M13 phage grafted with peptide motifs as a tool to detect amyloid-β oligomers in brain tissue
Source: Commun Biol. 2024 Jan 27;7:134. doi: 10.1038/s42003-024-05806-5 (PMC10821927; doi:10.1038/s42003-024-05806-5)
Supplement: Supplementary file 5 — Reporting summary [file 42003_2024_5806_MOESM5_ESM.pdf]

nature portfolio

Corresponding author(s): Helmut Kessels & Ivone Martins; COMMSBIO-23-1008A_1701722193_25

Last updated by author(s): 2023/12/15

Reporting Summary

Nature Portfolio wishes to improve the reproducibility of the work that we publish. This form provides structure for consistency and transparency in reporting. For further information on Nature Portfolio policies, see our Editorial Policies and the Editorial Policy Checklist.

Statistics

For all statistical analyses, confirm that the following items are present in the figure legend, table legend, main text, or Methods section.

n/a	Confirmed	
☐	☒	The exact sample size (n) for each experimental group/condition, given as a discrete number and unit of measurement
☐	☒	A statement on whether measurements were taken from distinct samples or whether the same sample was measured repeatedly
☐	☒	The statistical test(s) used AND whether they are one- or two-sided<br>*Only common tests should be described solely by name; describe more complex techniques in the Methods section.*
☒	☐	A description of all covariates tested
☒	☐	A description of any assumptions or corrections, such as tests of normality and adjustment for multiple comparisons
☐	☒	A full description of the statistical parameters including central tendency (e.g. means) or other basic estimates (e.g. regression coefficient) AND variation (e.g. standard deviation) or associated estimates of uncertainty (e.g. confidence intervals)
☐	☒	For null hypothesis testing, the test statistic (e.g. F, t, r) with confidence intervals, effect sizes, degrees of freedom and P value noted<br>*Give P values as exact values whenever suitable.*
☒	☐	For Bayesian analysis, information on the choice of priors and Markov chain Monte Carlo settings
☒	☐	For hierarchical and complex designs, identification of the appropriate level for tests and full reporting of outcomes
☒	☐	Estimates of effect sizes (e.g. Cohen's d, Pearson's r), indicating how they were calculated

Our web collection on statistics for biologists contains articles on many of the points above.

Software and code

Policy information about availability of computer code

Data collection	n/a
Data analysis	ImageJ was used to quantify fluorescence signal in brain slices.

For manuscripts utilizing custom algorithms or software that are central to the research but not yet described in published literature, software must be made available to editors and reviewers. We strongly encourage code deposition in a community repository (e.g. GitHub). See the Nature Portfolio guidelines for submitting code & software for further information.

Data

Policy information about availability of data

All manuscripts must include a data availability statement. This statement should provide the following information, where applicable:

- Accession codes, unique identifiers, or web links for publicly available datasets
- A description of any restrictions on data availability
- For clinical datasets or third party data, please ensure that the statement adheres to our policy

All data supporting the findings of this study are available within the paper and its Supplementary Information.
Should any raw data files be needed in another format they are available from the corresponding author upon reasonable request.

Human research participants

Policy information about studies involving human research participants and Sex and Gender in Research.

Reporting on sex and gender	n/a
Population characteristics	n/a
Recruitment	n/a
Ethics oversight	n/a

Note that full information on the approval of the study protocol must also be provided in the manuscript.

Field-specific reporting

Please select the one below that is the best fit for your research. If you are not sure, read the appropriate sections before making your selection.

☒ Life sciences ☐ Behavioural & social sciences ☐ Ecological, evolutionary & environmental sciences

For a reference copy of the document with all sections, see nature.com/documents/nr-reporting-summary-flat.pdf

Life sciences study design

All studies must disclose on these points even when the disclosure is negative.

Sample size	Sample sizes were determined by a power analysis based on preliminary data.
Data exclusions	No data was excluded from the analysis.
Replication	Experimental findings were reproduced multiple times in the study and all attempts at replications were successful. Furthermore, experimental conditions were described with detail to allow reproducibility and distinguish biological and technical replicates.
Randomization	Animal selection either by genotype or age was performed randomly.
Blinding	Data were analyzed by experimenter who was blinded for the genotypes and age of the mice.

Reporting for specific materials, systems and methods

We require information from authors about some types of materials, experimental systems and methods used in many studies. Here, indicate whether each material, system or method listed is relevant to your study. If you are not sure if a list item applies to your research, read the appropriate section before selecting a response.

Materials & experimental systems

n/a	Involved in the study	
☐	☒	Antibodies
☒	☐	Eukaryotic cell lines
☒	☐	Palaeontology and archaeology
☐	☒	Animals and other organisms
☒	☐	Clinical data
☒	☐	Dual use research of concern

Methods

n/a	Involved in the study	
☒	☐	ChIP-seq
☒	☐	Flow cytometry
☒	☐	MRI-based neuroimaging

Antibodies

Antibodies used	Primary antibodies: rabbit anti-fd bacteriophage antibody (Sigma, B7786, polyclonal); mouse anti-β-amyloid 6E10 antibody (BioLegend, 803001, monoclonal) and anti-amyloid fibrils OC antibody (AB2286, Merck, monoclonal).<br>Secundary antibodies: fluoresceinisothiocyanate (FITC)-labeled goat anti-rabbit IgG antibody (Sigma, F9887, polyclonal); donkey antiMouse IgG (H+L) labeled with Alexa Fluor 594 (Thermo Scientific, A-21203, polyclonal).
Validation	Rabbit anti-fd bacteriophage antibody: Synchronous selection of homing peptides for multiple tissues by in vivo phage display. Mikhail G Kolonin et al. FASEB journal: official publication of the Federation of American Societies for Experimental Biology, 20(7),

979-981 (2006-04-04).
Mouse anti-β-amyloid 6E10 antibody: Sandoval-Hernández A, et al. 2015. PLoS One. 10: 0145467.

Animals and other research organisms

Policy information about studies involving animals; ARRIVE guidelines recommended for reporting animal research, and Sex and Gender in Research

Laboratory animals	APPswe/PS1dE9-transgenic mice and wild-type c57bl/6 controls.
Wild animals	n/a
Reporting on sex	Study groups were formed with both male and female mice.
Field-collected samples	n/a
Ethics oversight	All experiments were conducted in line with the European guidelines for the care and use of laboratory animals (Council Directive 86/6009/EEC). The experimental protocol was approved by the Animal Experiment Committee of University of Amsterdam.

Note that full information on the approval of the study protocol must also be provided in the manuscript.
